# Development of an Artificial Intelligence-Based Chromosome Interpretation System for Amniotic Fluid Karyotyping

**DOI:** 10.3390/ijms27041746

**Published:** 2026-02-11

**Authors:** Kuan-Han Wu, Hsuan-Wei Huang, Chia Yun Lin, Hsu-Tung Huang, Tzuo-Yau Fan, Yueh-Peng Chen, Yung-Chiao Chang, Te-Yao Hsu, Kuo-Chung Lan

**Affiliations:** 1 Department of Emergency Medicine, Kaohsiung Chang Gung Memorial Hospital, Chang Gung University College of Medicine, Kaohsiung 833, Taiwan; hayatowu1120@gmail.com; 2 Department of Obstetrics and Gynecology, Jen-Ai Hospital, Taichung 483, Taiwan; yrsam8871@gmail.com (H.-W.H.); kidsshine@cgmh.org.tw (C.Y.L.); 3 Department of Obstetrics and Gynecology, Kaohsiung Chang Gung Memorial Hospital, Chang Gung University College of Medicine, Kaohsiung 833, Taiwan; hsutunghuang@outlook.com (H.-T.H.); maurenlab15@gmail.com (Y.-C.C.); tyhsu@cgmh.org.tw (T.-Y.H.); 4 Department of Obstetrics and Gynecology, Chiayi Chang Gung Memorial Hospital, Chang Gung University College of Medicine, Chiayi 613, Taiwan; 5 Center for Artificial Intelligence in Medicine, Linkou Chang Gung Memorial Hospital and Master of Science Degree Program in Innovation for Smart Medicine, Chang Gung University, Taoyuan 333, Taiwan; yaufan0625@gmail.com (T.-Y.F.); yuepengc@gmail.com (Y.-P.C.); 6 Department of Research and Development, Chang Gung Medical Technology Co., Ltd., Taoyuan 333, Taiwan; 7 Division of Rheumatology, Allergy and Immunology, Linkou Chang Gung Memorial Hospital, Taoyuan 333, Taiwan; 8 School of Medicine, College of Medicine, National Sun Yat-sen University, Kaohsiung 804, Taiwan

**Keywords:** amniotic fluid, chromosome segmentation, artificial intelligence, deep learning, segmentation, karyotype, cytogenetics

## Abstract

Conventional G-banded karyotyping remains indispensable in prenatal diagnosis but continues to rely on labor-intensive, expertise-dependent visual examination. To address these challenges, we developed a modular artificial intelligence (AI) workflow that automates chromosome interpretation from amniotic fluid metaphase images. The system integrates image denoising, chromosome segmentation, overlap screening, and morphology-based classification, and was trained using 13,223 clinical cases comprising more than 50,000 manually annotated chromosomes. Across training, temporal validation, and independent testing cohorts, classification accuracy remained consistently high (97.45%, 96.95%, and 95.72%, respectively). The overlap-recognition module further reduced downstream errors by reliably identifying composite chromosome regions. When applied to unsorted metaphase images from a later clinical cohort, the workflow successfully generated draft karyotypes without manual sorting and maintained close concordance with expert review. These findings demonstrate that an AI-assisted pipeline can support cytogenetic laboratories by streamlining the most labor-intensive steps of karyotyping, potentially enhancing diagnostic efficiency while preserving interpretive reliability.

## 1. Introduction

Amniocentesis remains a cornerstone of prenatal diagnosis by enabling direct cytogenetic analysis of fetal chromosomes through conventional G-banded karyotyping [1,2]. Despite advances in molecular techniques and non-invasive prenatal testing (NIPT), karyotyping continues to play an indispensable role in detecting numerical abnormalities, balanced structural rearrangements, and large-scale chromosomal alterations that are not reliably identified by sequencing-based approaches [3,4]. However, manual karyotype interpretation is labor-intensive, time-consuming, and highly dependent on the expertise of trained cytogeneticists, creating increasing challenges in routine clinical practice [5].

Early attempts to automate chromosome analysis focused primarily on idealized metaphase spreads, often assuming complete sets of well-separated chromosomes [6]. These systems typically relied on handcrafted features or early artificial neural network classifiers and demonstrated limited robustness when confronted with overlapping chromosomes, variable staining quality, or structural abnormalities commonly encountered in prenatal specimens [7]. More recent artificial intelligence-based pipelines, including CNN-based detection frameworks such as Mask-R-CNN-derived models used for chromosome segmentation and deep learning enumeration frameworks such as DeepACEv2 [8], have shown promising performance under controlled experimental conditions. Nevertheless, many of these approaches depend on pre-segmented chromosomes or assume ideal metaphase preparations, limiting their applicability to routine prenatal datasets characterized by heterogeneous image quality and frequent chromosome overlap [9].

Given these limitations, artificial intelligence offers an opportunity to streamline chromosome interpretation while reducing manual workload under real-world clinical conditions [10]. In this study, we present an end-to-end AI-assisted workflow designed for chromosome interpretation from amniotic fluid metaphase images obtained during routine clinical practice. Rather than relying on a single artificial neural network for generic image recognition, the proposed system adopts a modular design that integrates chromosome segmentation, overlap recognition, morphology-based classification, and draft karyotype assembly. This design enables transparent processing at each stage and facilitates expert review within routine prenatal cytogenetic workflows.

## 2. Results

### 2.1. Classification Accuracy Across Datasets

The chromosome-classification model was trained and evaluated using temporally separated datasets collected over multiple years. Classification accuracy remained consistently high across the training, validation, and independent testing cohorts, reaching 97.45%, 96.95%, and 95.72%, respectively (Table 1). These results indicate stable classification performance across datasets acquired under different time periods and routine laboratory conditions.

### 2.2. Performance Metrics for Individual Chromosome Classes

Detailed performance metrics for individual chromosome classes are summarized in Table 2, allowing per-class evaluation of the model’s behavior beyond overall accuracy. Across most chromosome classes, both PPV and NPV remained high, indicating reliable identification of target chromosomes and effective exclusion of non-target classes. Performance variations were primarily observed among chromosomes with similar morphology and G-banding patterns.

### 2.3. Error Distribution and Confusion Matrix Analysis

**Figure 1:**
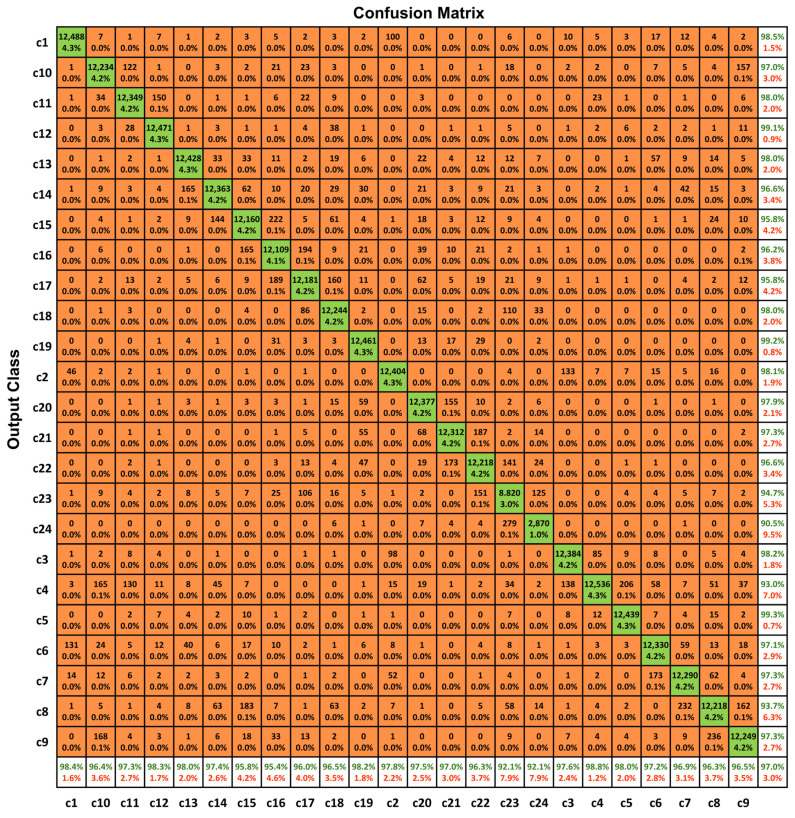
Confusion matrix of chromosome classification results obtained from the independent testing cohort. Rows represent the true chromosome classes, and columns represent the predicted classes. Diagonal elements indicate correctly classified chromosomes, whereas off-diagonal elements correspond to misclassifications between chromosome classes.

Analysis of the confusion matrix (Figure 1) revealed that classification errors were not randomly distributed but were predominantly confined to morphologically similar chromosome classes. Most misclassifications occurred between neighboring autosomes with comparable size and G-banding patterns, such as chromosomes 14 and 15 or chromosomes 21 and 22. In contrast, large or structurally distinct chromosomes demonstrated consistently high classification accuracy.

Importantly, no systematic bias toward over- or under-prediction of specific chromosome classes was observed, as reflected by the dominance of diagonal elements across the matrix. These error patterns closely mirror the challenges encountered during manual karyotype interpretation and suggest that the observed misclassifications reflect biologically plausible ambiguities rather than systematic classification failure.

### 2.4. Overlap Recognition Performance

The overlap-recognition module was evaluated to determine its ability to distinguish between isolated chromosomes and overlapping chromosome regions. The model accurately identified overlapping regions across a range of metaphase images, enabling effective filtering of ambiguous chromosome segments prior to downstream classification. This performance supports reliable handling of complex metaphase images containing overlapping chromosomes.

### 2.5. Application to Unsorted Metaphase Images

**Figure 2:**
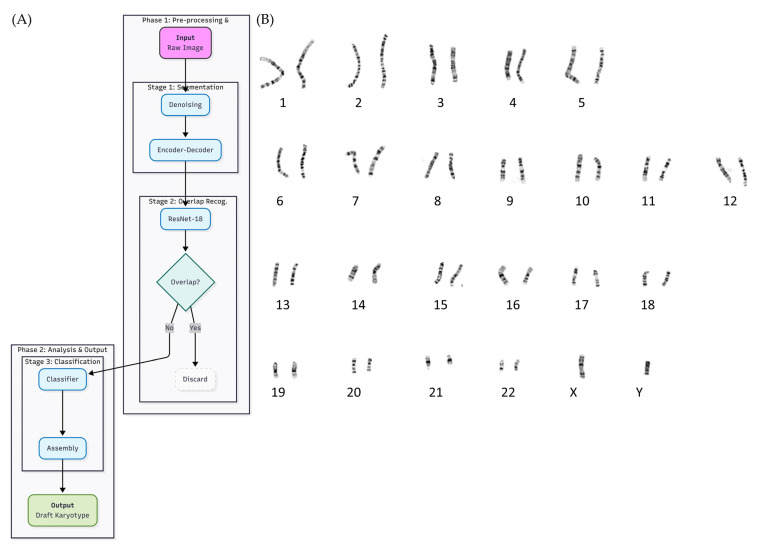
Automated inference workflow applied to unsorted metaphase images. (**A**) Overall schematic of the AI-assisted karyotyping pipeline, consisting of image denoising and segmentation (Stage 1), overlap filtering using a ResNet-18 module to remove ambiguous or overlapping chromosome regions (Stage 2), and morphology-based classification and draft karyotype assembly (Stage 3). (**B**) Representative examples of chromosome segmentation and overlap recognition, showing raw chromosome regions, refined segmentation masks, and identified overlapping regions removed prior to classification.

To evaluate real-world applicability, the complete AI workflow was applied to an independent set of unsorted G-banded metaphase images. As shown in Figure 2, the system processes raw metaphase inputs without manual preselection and executes the same three-stage pipeline used during model development.

In Stage 1, raw metaphase images undergo denoising followed by automated extraction of chromosome-containing regions using a convolutional encoder–decoder segmentation network. In Stage 2, each segmented region is evaluated by a ResNet-18-based overlap-recognition module, which removes overlapping or composite chromosome structures to prevent downstream misclassification. Representative examples of segmentation refinement and overlap detection are shown in Figure 2B.

In Stage 3, isolated chromosomes are classified into 24 morphological categories and automatically assembled into a draft karyotype. When applied to unsorted clinical metaphase images, the workflow reliably extracted individual chromosomes, filtered ambiguous regions, and generated draft karyotypes consistent with expert interpretations. These results demonstrate that the proposed system can operate effectively on uncurated metaphase images and is suitable for routine prenatal cytogenetic practice.

## 3. Discussion

This study presents an AI-assisted workflow designed to support the interpretation of G-banded chromosomes from amniotic fluid samples in routine prenatal cytogenetics. By integrating chromosome segmentation, overlap recognition, morphology-based classification, and draft karyotype assembly, the proposed system aims to reduce the manual effort required for chromosome sorting while preserving expert oversight throughout the diagnostic process.

Across temporally separated cohorts collected over multiple years, the chromosome-classification module demonstrated consistently high performance under routine laboratory conditions. Although classification accuracy decreased modestly from the training to the independent testing cohort, this trend likely reflects increased heterogeneity in staining quality, chromosome spreading, and overlap patterns commonly encountered in real-world prenatal datasets. Importantly, most misclassifications occurred among chromosomes with similar size and banding characteristics, mirroring challenges faced during manual karyotype interpretation and suggesting that the observed errors were morphologically plausible rather than systematic.

In the context of prenatal cytogenetics, the system is explicitly intended to function as a clinical decision-support tool rather than an autonomous diagnostic platform. From this perspective, performance metrics such as positive predictive value (PPV) and negative predictive value (NPV) provide particularly relevant information. High PPV indicates that chromosomes assigned to a given class are likely to be correct, thereby reducing the burden of manual verification, whereas high NPV helps ensure that true homologs are not inadvertently excluded during karyotype assembly. Together, these metrics align closely with routine laboratory workflows and expert review practices.

The incorporation of an overlap-recognition module represents a key design feature of the proposed workflow. Overlapping chromosomes are a frequent source of ambiguity in automated cytogenetic analysis, as segmentation errors can propagate into downstream classification. By identifying and filtering overlapping regions prior to classification, the workflow limits such error propagation and supports more reliable draft karyotype assembly. This modular strategy differs from earlier automated karyotyping systems that assumed idealized metaphase spreads and lacked mechanisms to address structural complexity in prenatal samples.

Compared with previously reported AI-based chromosome-analysis pipelines, which often rely on pre-segmented inputs or controlled image conditions [11], the present study emphasizes applicability to unsorted metaphase images obtained during routine clinical practice. The ability to generate draft karyotypes directly from these images represents an important step toward practical integration into prenatal cytogenetic workflows, where initial image quality and chromosome arrangement are highly variable.

Several limitations of this study should be acknowledged. First, all data were derived from a single institution, and performance may vary under different staining protocols, imaging systems, or laboratory practices. Second, although the dataset was large, rare structural abnormalities were underrepresented, and additional data will be required to fully assess system performance in these cases. Finally, the workflow does not independently diagnose chromosomal abnormalities but instead provides draft karyotypes that require expert verification.

Overall, this study demonstrates that an AI-assisted workflow can meaningfully enhance the efficiency of prenatal cytogenetic analysis without replacing expert interpretation. By streamlining the most labor-intensive steps of karyotyping and supporting expert review, such systems may help address increasing clinical workloads while maintaining diagnostic reliability.

## 4. Materials and Methods

### 4.1. Data Sources

Metaphase images were collected from 13,223 amniotic fluid samples processed at the Cytogenetics Laboratory of Kaohsiung Chang Gung Memorial Hospital (CGMH) between 2014 and 2020. The dataset included both normal and abnormal karyotypes encountered in routine prenatal diagnosis. For model development, the images were divided into separate temporal cohorts for training (2014–2015), validation (2016), and independent testing (2017–2018). Additional cases collected in 2019 were used for workflow evaluation with unsorted metaphase images. The number of cases and images included in each cohort is summarized in Table 3. All samples were anonymized before analysis.

### 4.2. Chromosome Annotation

Chromosomes were manually annotated by trained cytogeneticists to generate ground-truth labels for supervised learning. For each metaphase image, individual chromosomes were delineated using polygonal masks or bounding boxes. Regions containing overlapping or entangled chromosome structures were additionally annotated and labeled as overlapping regions.

In total, more than 50,000 chromosome instances across 24 biologically defined classes (22 autosomes and the X and Y chromosomes) were curated. These annotations were used for training and validating the segmentation, overlap-recognition, and chromosome-classification modules.

### 4.3. Model Components

#### 4.3.1. Denoising Module

Raw G-banded metaphase images were first subjected to image denoising to reduce background noise and enhance chromosome contours. This preprocessing step aimed to improve image quality prior to segmentation by generating clearer chromosome boundaries and suppressing staining artifacts.

#### 4.3.2. Segmentation Module

Chromosome segmentation was performed to extract candidate chromosome regions from denoised metaphase images. A convolutional neural network-based segmentation model was trained to distinguish chromosome foreground from background regions. Morphological postprocessing was subsequently applied to refine chromosome edges and remove residual artifacts.

**Figure 3:**
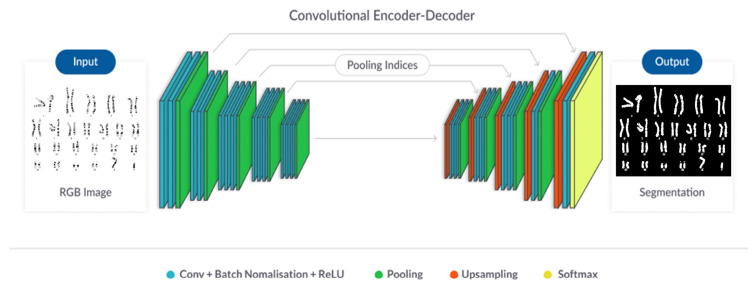
Overview of the chromosome segmentation module. The figure illustrates the encoder–decoder architecture used to extract chromosome regions from raw G-banded metaphase images. Intermediate feature maps and post-processing steps are shown to demonstrate how single-chromosome candidates are isolated prior to overlap recognition.

Representative examples of the chromosome segmentation process, including raw inputs, segmentation masks, and refined outputs, are shown in Figure 3.

#### 4.3.3. Overlap Recognition Module

To resolve ambiguities caused by overlapping or closely apposed chromosomes, an overlap-recognition module was developed to distinguish isolated chromosomes from overlapping chromosome structures. Separate annotated datasets were curated specifically for this task: 17,000 regions containing true overlapping chromosomes and 17,000 regions containing single, non-overlapping chromosomes. These regions were generated from the segmentation output and manually reviewed by trained cytogenetic technologists to ensure high-quality labeling.

A ResNet-18-based binary classifier was trained on this dataset to output the probability that a segmented region represents either a single chromosome or an overlapping structure. This module operates as a mandatory quality-control gate within the overall workflow, filtering out ambiguous regions before they proceed to downstream analysis.

**Figure 4:**
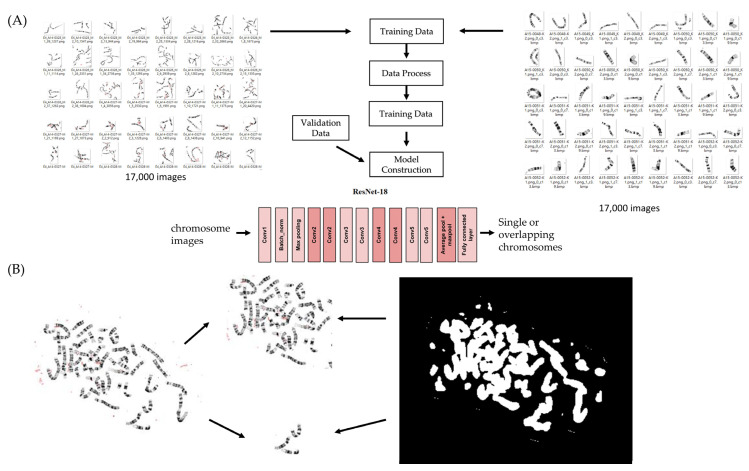
Overlap-recognition module and training workflow. (**A**) Overview of the dataset preparation and model development process for overlap recognition. Separate annotated datasets of overlapping and non-overlapping chromosome regions were used to train and validate the ResNet-18-based module. (**B**) Architecture and representative outputs of the overlap-recognition model. The ResNet-18 network receives segmented chromosome regions and predicts whether each region contains a single chromosome or overlapping chromosomes, enabling exclusion of ambiguous regions prior to downstream processing.

The training structure of this module, its integration within the multi-stage pipeline, and representative examples of its output categories are shown in Figure 4, which illustrates both the dataset construction workflow (Panel A) and the ResNet-18 architectural schematic with output behavior (Panel B).

#### 4.3.4. Classification Module

Segmented chromosome regions identified as isolated were subsequently processed by the chromosome-classification module. Each chromosome region was classified into one of 24 biologically defined categories corresponding to the 22 autosomes and the X and Y chromosomes.

The classification model output a predicted chromosome class together with an associated confidence score. These outputs were later used during draft karyotype assembly for expert review rather than for autonomous diagnosis.

### 4.4. Performance Evaluation

Model performance was evaluated at the individual-chromosome-instance level. Each segmented chromosome was treated as an independent instance, and classification accuracy was defined as the proportion of correctly classified chromosome instances among all evaluated instances within each dataset.

Evaluation metrics included sensitivity, specificity, precision, recall, F1 score, positive predictive value (PPV), and negative predictive value (NPV). These metrics were calculated as follows: sensitivity = TP(TP + FN); specificity = TN/(TN + FP); precision (PPV) = TP/(TP + FP); recall = TP/(TP + FN); NPV = TN/(TN + FN); and F1 score = 2 × (precision × recall)/(precision + recall). TP, FP, TN, and FN denote true positives, false positives, true negatives, and false negatives, respectively.

Overall classification accuracy across datasets is summarized in Table 1, and detailed per-class performance metrics are provided in Table 2.

### 4.5. System Overview

The proposed system follows a modular end-to-end workflow for chromosome interpretation from amniotic fluid metaphase images. The workflow consists of chromosome segmentation, overlap recognition, morphology-based chromosome classification, and draft karyotype assembly. Each module was trained independently using dedicated annotated datasets, enabling transparent processing and evaluation at each stage.

## 5. Conclusions

This study presents an artificial intelligence-assisted workflow that supports conventional G-banded karyotyping in prenatal cytogenetics. By integrating chromosome segmentation, overlap recognition, and morphology-based classification, the system reduces the need for manual chromosome sorting and addresses common sources of error in routine analysis.

The workflow achieved consistently high classification accuracy across multiple real-world clinical cohorts and successfully generated draft karyotypes from unsorted metaphase images that closely matched expert interpretation. Rather than operating as an autonomous diagnostic tool, the system functions as a clinical decision-support aid, improving efficiency while preserving expert oversight.

## Figures and Tables

**Table 1:** Classification accuracy of the chromosome morphology classifier across the training, validation, and independent testing cohorts.

Data Set	Year	C1–C22	C23	C24	Accuracy
Training Data	2014/03–2015/12	17,408 × 22	13,269	4358	97.45%
Validation Data	2016/01–2016/12	12,688 × 22	9573	3115	96.95%
Testing Data	2017/01–2018/02	21,010 × 22	15,906	5194	95.72%

**Table 2:** Classification performance metrics for each of the 24 chromosome classes in the independent testing set.

Type	TP	FP	TN	FN	Sensitivity	Specificity	Precision	Recall	F1	PPV	NPV
1	992	20	22,980	8	0.992	0.999	0.98	0.992	0.986	0.98	1
2	986	16	22,984	14	0.986	0.999	0.984	0.986	0.985	0.984	0.999
3	987	12	22,988	13	0.987	0.999	0.988	0.987	0.987	0.988	0.999
4	988	58	22,942	12	0.988	0.997	0.945	0.988	0.966	0.945	0.999
5	988	14	22,986	12	0.988	0.999	0.986	0.988	0.987	0.986	0.999
6	976	23	22,977	24	0.976	0.999	0.977	0.976	0.976	0.977	0.999
7	976	22	22,978	24	0.976	0.999	0.978	0.976	0.977	0.978	0.999
8	969	39	22,961	31	0.969	0.998	0.961	0.969	0.965	0.961	0.999
9	977	31	22,969	23	0.977	0.999	0.969	0.977	0.973	0.969	0.999
10	971	21	22,979	29	0.971	0.999	0.979	0.971	0.975	0.979	0.999
11	973	13	22,987	27	0.973	0.999	0.987	0973	0.98	0.987	0.999
12	987	7	22,993	13	0.987	1	0.993	0.987	0.99	0.993	0.999
13	983	34	22,966	17	0.983	0.999	0.967	0.983	0.975	0.967	0.999
14	975	39	22,961	25	0.975	0.998	0.962	0.975	0.68	0.962	0.999
15	958	42	22,958	42	0.958	0.998	0.958	0.958	0.958	0.958	0.998
16	965	37	22,963	35	0.965	0.998	0.963	0.965	0.964	0.963	0.998
17	961	36	22,964	39	0.961	0.998	0.964	0.961	0.962	0.964	0.998
18	967	27	22,973	33	0.967	0.999	0.973	0.967	0.97	0.973	0.999
19	985	11	22,989	15	0.985	1	0.989	0.985	0.987	0.989	0.999
20	983	20	22,980	17	0.983	0.999	0.98	0.983	0.982	0.98	0.999
21	974	18	22,982	26	0.974	0.999	0.982	0.974	0.978	0.982	0.999
22	962	31	22,969	38	0.962	0.999	0.969	0.962	0.965	0.969	0.998
23	916	59	22,941	84	0.916	0.997	0.939	0.916	0.928	0.939	0.996
24	938	33	22,967	62	0.938	0.999	0.966	0.938	0.952	0.966	0.997

Abbreviations: TP, True Positive; TN, True Negative; FP, False Positive; FN, False Negative; PPV, Positive Predictive Value; NPV, Negative Predictive Value.

**Table 3:** Distribution of amniotic fluid samples across study cohorts used for model development and evaluation.

Year	Patients	Images (K)	Images (M)	Male	Female	Abnormalities	Missing Reports
2014	2117	4135	4321	1062	1055	27	117
2015	2717	5507	5506	1406	1311	37	0
2016	3141	6366	6365	1613	1528	38	8
2017	2815	5719	5725	1414	1401	37	13
2018	2433	4951	4959	1260	1173	37	31
Total	13,223	26,858	26,876	6755	6468	176	169

Note: The dataset includes both sorted and unsorted images. The number of karyogram images (K) corresponds to the chromosomal karyotypes, while the number of metaphase spread images (M) represents the scattered chromosome arrangements during metaphase.

## Data Availability

The data generated/analyzed in this study are available from the corresponding author upon request.
